# The Role of Wnt Signaling in Postnatal Tooth Root Development

**DOI:** 10.3389/fdmed.2021.769134

**Published:** 2021-11-12

**Authors:** Nicha Tokavanich, Marc N. Wein, Jeryl D. English, Noriaki Ono, Wanida Ono

**Affiliations:** 1 Endocrine Unit, Massachusetts General Hospital and Harvard Medical School, Boston, MA, United States; 2 Department of Developmental Biology, Harvard School of Dental Medicine, Boston, MA, United States; 3 Department of Orthodontics, University of Texas Health Science Center at Houston School of Dentistry, Houston, TX, United States; 4 Department of Diagnostic and Biomedical Sciences, University of Texas Health Science Center at Houston School of Dentistry, Houston, TX, United States

**Keywords:** Wnt, signaling pathway, tooth eruption, tooth root formation, tooth development

## Abstract

Appropriate tooth root formation and tooth eruption are critical for achieving and maintaining good oral health and quality of life. Tooth eruption is the process through which teeth emerge from their intraosseous position to their functional position in the oral cavity. This temporospatial process occurs simultaneously with tooth root formation through a cascade of interactions between the epithelial and adjoining mesenchymal cells. Here, we will review the role of the Wnt system in postnatal tooth root development. This signaling pathway orchestrates the process of tooth root formation and tooth eruption in conjunction with several other major signaling pathways. The Wnt signaling pathway is comprised of the canonical, or Wnt/β-catenin, and the non-Canonical signaling pathway. The expression of multiple Wnt ligands and their downstream transcription factors including β-catenin is found in the cells in the epithelia and mesenchyme starting from the initiation stage of tooth development. The inhibition of canonical Wnt signaling in an early stage arrests odontogenesis. Wnt transcription factors continue to be present in dental follicle cells, the progenitor cells responsible for differentiation into cells constituting the tooth root and the periodontal tissue apparatus. This expression occurs concurrently with osteogenesis and cementogenesis. The conditional ablation of β-catenin in osteoblast and odontoblast causes the malformation of the root dentin and cementum. On the contrary, the overexpression of β-catenin led to shorter molar roots with thin and hypo-mineralized dentin, along with the failure of tooth eruption. Therefore, the proper expression of Wnt signaling during dental development is crucial for regulating the proliferation, differentiation, as well as epithelial-mesenchymal interaction essential for tooth root formation and tooth eruption.

## INTRODUCTION

Proper tooth root formation and tooth eruption are crucial for achieving lifelong oral and overall health, as it directly impacts fundamental human functions, including mastication and speech. The dental follicle, a loose sac-like structure surrounding the tooth bud, contains mesenchymal progenitor cells that play a crucial part in these processes; it contains precursor cells for cementoblasts, periodontal ligament cells, and cryptal bone osteoblasts, therefore, it serves as a critical component of tooth root formation. Tooth root formation starts after the dental crown formation. In the late bell stage, the inner and outer enamel epithelial fuse together and form Hertwig’s epithelial root sheath (HERS). The HERS lies between the dental papilla and dental follicle (1). The interaction of the HERS and the surrounding neural crest derived-mesenchymal cells is required for the formation of the tooth root (2-5). The HERS serves as a guide for tooth root formation, and eventually disintegrates and becomes the epithelial rests of Malassez (ERM) (6). The cells in the dental papilla adjacent to HERS differentiate into odontoblasts and generate the root dentin (7). These highly dynamic epithelial-mesenchymal interactions involve major signaling cascades, including the Wnt signaling pathway.

Tooth eruption is driven by the coronal bone resorption induced by the dental follicle and the force generated by the elongation of the tooth root (8). Dental follicle cells also play a major role in the tooth-eruption process by recruiting osteoclasts to create the path of eruption and differentiate themselves into cells constituting the periodontal tissue surrounding the tooth root. The process of tooth eruption is defined as the movement of teeth from their intraosseous developmental site to their functional position in the oral cavity (9-11). Tooth eruption is achieved through three distinct steps: (a) pre-eruptive tooth movement, (b) eruptive tooth movement, and (c) post-eruptive tooth movement (4, 12, 13). This process occurs simultaneously with the formation of the tooth root and is orchestrated by networks of signaling pathways, including Wnt signaling, parathyroid/parathyroid-related protein (PTHrP), bone morphogenetic protein (BMP), sonic hedgehog (Shh), and transforming growth factor-beta (TGF-β) (2, 14-19).

The Wnt signaling pathway is widely known to play an essential role in multiple stages of tooth development, starting from the initiation of the crown formation to the completion of the tooth root formation. Furthermore, Wnt signaling is also involved in the tooth-eruption process. This review aims to outline the current knowledge on the roles of Wnt signaling in postnatal tooth development.

## WNT SIGNALING PATHWAY

**FIGURE 1 ∣:**
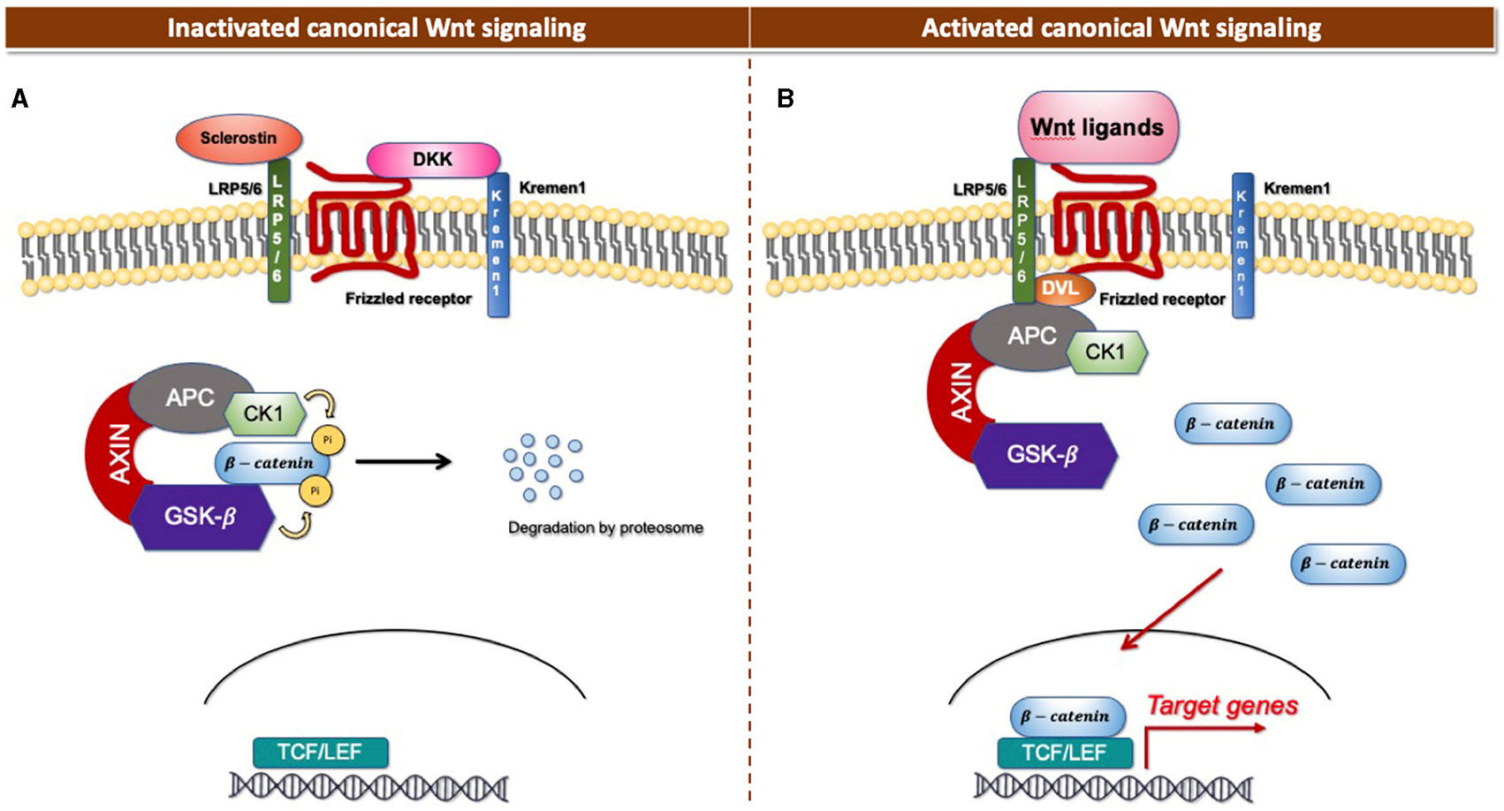
Canonical Wnt signaling. **(A)** Inactivated canonical Wnt signaling pathway. **(B)** Activated canonical Wnt signaling pathway.

**FIGURE 2 ∣:**
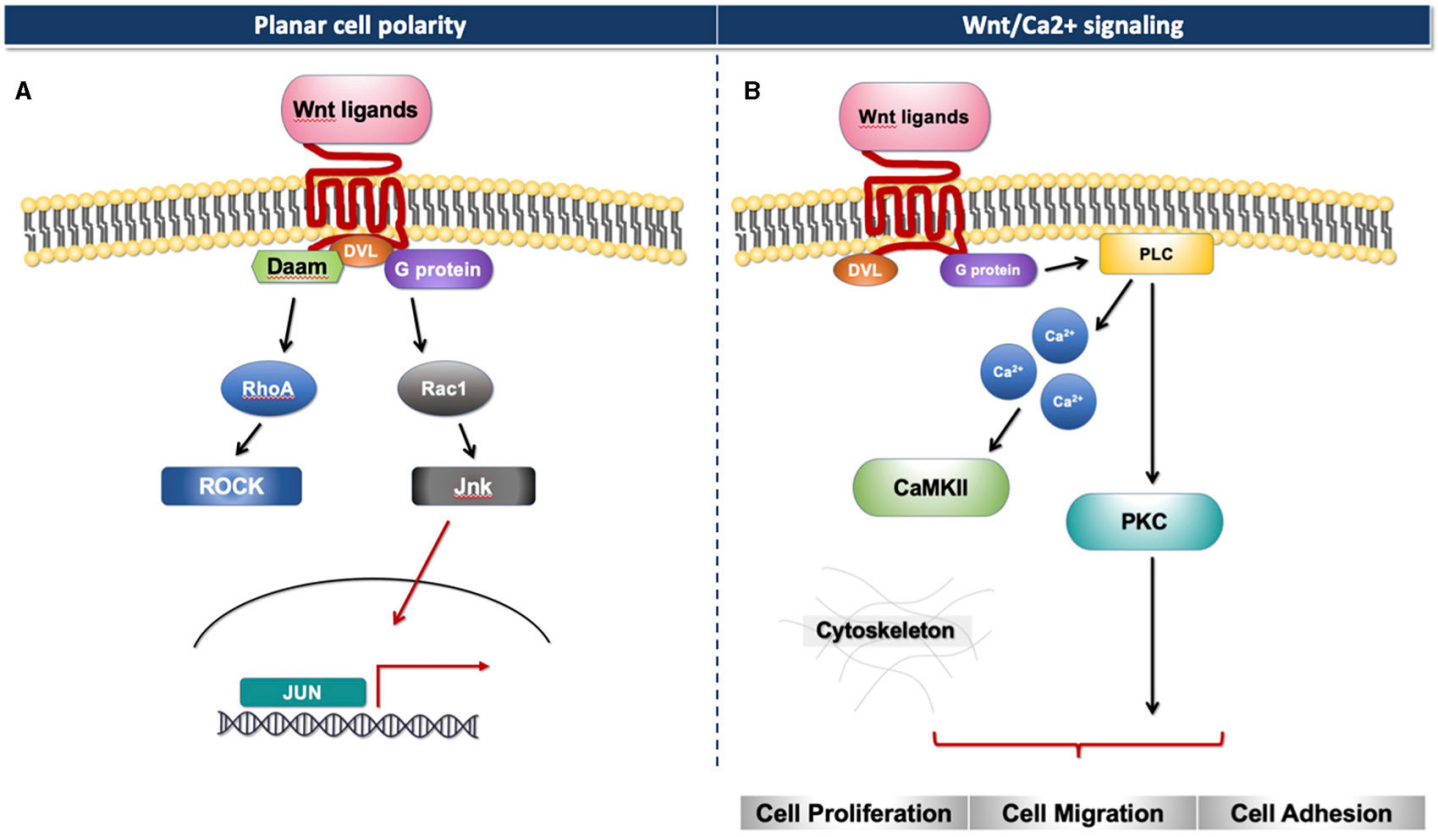
Non-canonical Wnt signaling. **(A)** Planar cell polarity signaling pathway. **(B)** Wnt/Ca2+ signaling pathway.

The Wnt signaling pathway consists of at least 19 Wnt secretory glycoproteins and is divided into two distinct pathways, the canonical or β-catenin-dependent pathway, and the non-canonical or β-catenin-independent pathway (20, 21). The canonical pathway regulates the function of cells through the intracellular β-catenin levels mediated by the Frizzled (FZD) receptors. In the absence of Wnt ligands, the cytoplasmic β-catenin binds to a protein complex comprised of adenomatous polyposis coli (APC), axin, and glycogen synthase kinase 3 (GSK3), then β-catenin is phosphorylated and targeted for degradation [Figure 1A; (22)]. In the presence of Wnt ligands, the ligands bind to an FZD receptor and inactivate the β-catenin degradation causing the accumulation of β-catenin that induces the translocation of cytoplasmic β-catenin to the nucleus, then further regulates the transcription of Wnt target genes [Figure 1B; (23)]. Non-canonical Wnt ligands, such as Wnt5a and Wnt11, trigger the β-catenin independent pathway. This pathway can be separated into two distinct tracts, including the Wnt/Planar cell polarity pathway (PCP) and the Wnt/Ca^2+^ signaling pathway (24). These signaling pathways play significant roles in tissue formation and homeostasis [Figure 2; (25)]. In addition, the non-canonical Wnt signaling pathway has been reported to modulate the canonical Wnt pathway in several ways. For example, Wnt5a can bind to the receptor tyrosine kinase (RTK)-like orphan receptor 2 (Ror2) which can inhibit or stimulate canonical Wnt signal at the transcriptional level of T-cell factor/lymphoid enhancer factor (TCF/LEF)-mediated (23, 26).

## WNT SIGNALING IN PRENATAL TOOTH DEVELOPMENT

Multiple canonical Wnt genes and their downstream transcription factors including Wnt4, Wnt5a, Wnt6, Wnt10a, Lef1 as well as β-catenin are spatiotemporally expressed in the dental epithelium and mesenchyme starting from the initiation stage of the crown development (27-33). In addition, other Wnt responsive genes, such as Axin2, are expressed in differentiating odontoblast (34). In addition, Wnt5a and Ror2, a non-canonical Wnt pathway ligand and receptor, are expressed in the prenatal dental epithelium and mesenchyme in the incisor and can be detected in the dental papilla of the molars (29, 31).

Studies have found that the Wnt/β-catenin signaling pathway plays an important role at multiple stages of tooth development (30). The inactivation of the canonical pathway in the dental epithelium and mesenchyme disrupts the tooth differentiation, resulting in arrests in odontogenesis in early tooth development (27, 30, 35-37). Moreover, the loss of Wnt/β-catenin signaling in the epithelial and mesenchymal cells in the later stage of the crown development impairs the molar cusps development, presenting flatten, small and irregular molar cusp morphology with an enlarged pulp (28, 30, 38). In contrast, the constitutive activation of the Wnt/β-catenin signaling pathway in the epithelium induces malformed and ectopic tooth formation, while the stabilization of this signaling pathway in the mesenchyme inhibits sequential tooth formation, resulting in the failure of the second and third molar development in tooth explant (28, 30, 39). Furthermore, the constitutive expression of β-catenin in mesenchymal cells accelerates dental pulp cells differentiation, resulting in premature odontoblasts with dentinlike materials formed within the dental pulp area (27). For the non-canonical Wnt signaling pathway, Wnt5a and Ror2 global deletion in mice results in delayed crown development, impaired odontoblast differentiation, and malformation of the crown, including blunt and absence of cusps found in the mutant molar as well as in the incisor. Thus, Wnt5a, along with Ror2, is important for tooth patterning and differentiation by regulating the Wnt/β-catenin signaling pathway (29).

In conclusion, the spatiotemporal activities of the canonical Wnt signaling are required to stimulate cell proliferation as well as the differentiation of dental epithelial and mesenchymal cells necessary for proper tooth development during the prenatal period. This pathway regulates the morphological patterns of the tooth crown which includes the number, form, size, and placement of teeth. The inactivation of the Wnt signaling pathway arrests tooth development. In contrast, the overexpression of the Wnt signaling pathway in epithelial cells induces ectopic tooth formation and premature cell differentiation. In addition, over-activation of Wnt signaling in mesenchymal cells impedes sequential tooth formation. The non-Canonical pathway may control tooth development and cell differentiation *via* regulating the canonical Wnt pathway.

## WNT SIGNALING IN TOOTH ROOT FORMATION

Tooth root formation occurs exclusively during the postnatal period in mice. Wnt/β-catenin signaling continues to be active in the dental epithelium (HERS) and the dental mesenchyme, including the cells in the dental papilla and the dental follicle adjacent to HERS (40, 41). Axin2, a negative regulator of the canonical Wnt pathway, is expressed in developing tooth roots starting from postnatal day (P) 10 around the HERS and the dental papilla (34). In addition, Wnt3a is expressed in the HERS and odontoblasts in 2-week-old mice and continues to be present in the epithelial rest cells of Malassez (42). Wnt10a expression can also be found in the epithelial and dental mesenchymal cells from prenatal throughout the tooth root formation (32, 43). Wnt5a and Ror2 continue to be expressed postnatally in the dental mesenchyme and epithelium. This expression pattern becomes more pronounced in the root-forming region over time. It has been reported that the Ror2-mediated signaling pathway in the dental mesenchyme modulates molar root formation through the activation of Cell Division Cycle 42 (Cdc42), the downstream target of the Ca^2+^ signaling pathway. This suggests the potential role of non-Canonical Wnt signaling in tooth root development (44).

Moreover, canonical Wnt signaling is found to crosstalk with other important signaling pathways to regulate normal tooth root formation. For example, the TGF-β signaling pathway, which is required for root odontogenesis. TGF-β is found to have a cooperative role with Wnt signaling. It is suggested that TGF-β might suppress canonical Wnt signaling to inhibit osteogenic potential and secures odontogenic potential in mesenchymal cells (34, 45).

**FIGURE 3 ∣:**
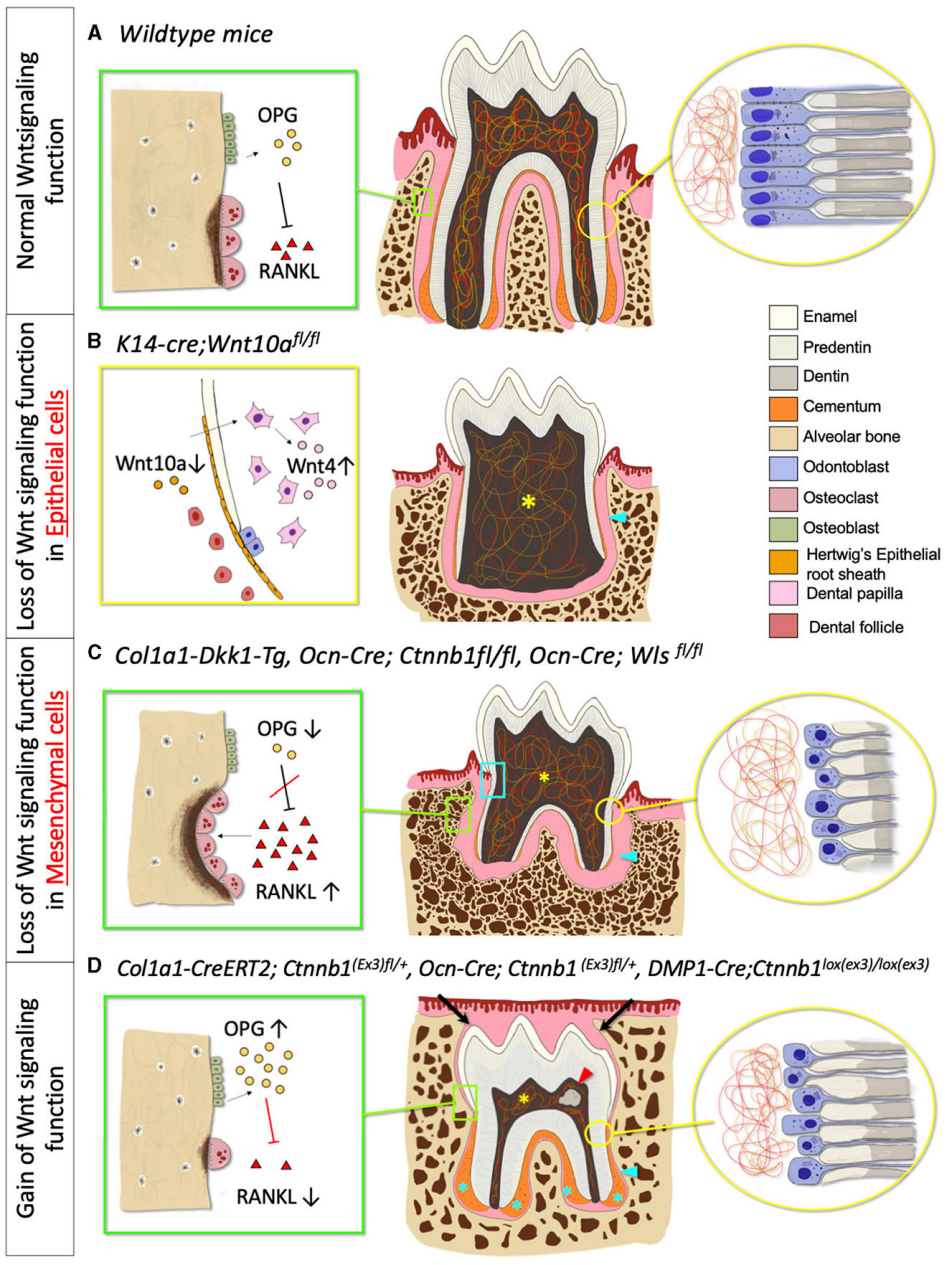
Phenotypic change in tooth morphology and tooth eruption in absent and excessive Wnt signaling function. **(A)** Normal Wnt signaling function phenotype. Green box: Osteoclast and osteoblast, Yellow circle: Odontoblast and dentin. **(B)** Loss of Wnt signaling in epithelial cells phenotype. Yellow box: Deletion of Wnt10a in HERS, increase Wnt4 expression in Dental papilla, Yellow asterisk: Lack of pulpal floor chamber, Blue arrowhead: Short and thin root dentin. **(C)** Loss of Wnt signaling in mesenchymal cells phenotype. Green box: Increased osteoclast activities and number, Yellow circle: Reduced odontoblast number and defective dentin, Blue box: Root resorption, Yellow asterisk: Enlarged pulpal chamber, Blue arrowhead: Short root with thinner root dentin, decreased cementoblasts number, and reduced cellular cementum. **(D)** Gain of Wnt signaling phenotype. Green box: Decreased osteoclast activities and number, Yellow circle: Premature odontoblast with hypo-mineralized dentin (increased predentin), Red arrowhead: Pulp stone, Blue asterisk: Hyper-cementosis with increased cementocyte number, Blue arrowhead: Short root with an increase in hypo-mineralized dentin, Black arrow: Delayed tooth eruption.

Wnt/β-catenin signaling has an important role in epithelial-mesenchymal interaction during tooth root development. The deletion of β-catenin in the epithelium using *Shh-creER* impairs the structural integrity of the HERS and prematurely disrupts the bilayer structure of HERS. This inactivation further interrupts odontogenesis with the reduced messenger RNA (mRNA) expression of *Osx*, *Nfic*, *Msx1*, and *Msx2*, and eventually, resulting in shorter molar roots. These findings suggest that β-catenin has an important role in modulating the structural integrity of HERS which is important for dentinogenesis and root development (46). Yu et al. also found that the selective ablation of Wnt10a in the epithelial cells (*K14-cre;Wnt10a^fl/fl^*) displays taurodontism with shortened root molars. The inactivation of Wnt10a in K14 expressed cells hinders epithelial cell proliferation and induces the compensatory elevation of Wnt4a in the dental papilla. This results in the excessive proliferation of dental papilla and eventually disrupts the pulp chamber floor formation. Interestingly, the deletion of Wnt10a in the dental mesenchyme results in normal molar formation [Figure 3B; (43)]. Changes in the β-catenin level have significant effects on the dental mesenchyme during postnatal tooth formation within a certain period (47). When β-catenin is deleted in the odontoblast lineage cells using Osteocalcin (*Ocn-cre; Ctnnb^fl/fl^*) during molar tooth root development, the differentiation and proliferation of odontoblasts are impeded, resulting in completely disrupted tooth root development. The phenotype manifests as a rootless molar with normal tooth eruption in the first and second molars (48, 49). Using the same promotor (*Ocn-cre; Wls^fl/fl^*), the conditional ablation of *Wntless* genes, which controls Wnt ligand trafficking and secretion, also hampers odontoblast differentiation and polarization, causing short molar roots with thin dentin and enlarged root canal (14). Correspondent with these studies, the inactivation of Wnt signaling by using Dickkopf 1 (DKK1) overexpression using a 2.3-kb Col1a1 driver (which targets odontoblasts and osteoblasts) exhibits the same short root phenotype. In addition, Dkk1 overexpression delays odontoblast maturation. As a result, the dentin formation is severely disrupted, and the dentinal tubules of mutant molars are less in number and disorganized associated with a significantly reduced rate of dentin formation [Figure 3C; (38)]. Furthermore, the specific ablation of Ror2 in the dental mesenchyme (*Osr2-Cre; Ror2^fl/fl^*) causes delayed tooth root formation with short and thick HERs formation, and the absence of furcation in mice molar at P12. At P17.5 the mutant mice exhibit shortened roots at the furcation region due to the mesenchymal hypo-proliferation and delayed odontogenesis during tooth root elongation. However, the deletion of Ror2 in the epithelial cells during tooth root formation does not affect the tooth root morphology nor cell differentiation, supporting the cell-type-specific function of Ror2 in dental mesenchymal cells (44).

Interestingly, the gain of the function mutation of β-catenin gene in the mesenchymal cells also exhibits disturbance in postnatal tooth root formation (Figure 3D). Wang et al. suggested that P0-P5 is a critical window of time in which the overexpression of β-catenin disturbs root elongation. The induction of excessive β-catenin using an inducible 3.2-kb *Col1-creER* transgenic line in P0 results in rootless molar and the phenotypes are less severe when tamoxifen is injected in later time points. Although this study showed that the regulation of root elongation is time-dependent, the defect in odontoblast differentiation and dentin secretion occurs at every induction time point, which indicates that excessive β-catenin affects the odontoblast differentiation and dentin formation in a continuous manner (47). The mutant mice with continuous stabilization of β-catenin in the dental mesenchyme, using *Ocn-cre* as a promoter (*Ocn-cre; Ctnnb*^*lox*(*ex*3)^), displayed short and distorted molar roots. The cell-specific β-catenin stabilization accelerates odontoblast differentiation, causing a thickened dentin with a hypo mineralized matrix (41, 50). The stabilization of β-catenin in the Col1a1-expressing cells (*Col1a1-cre; Ctnnb*^*lox*(*ex*3)^) also showed hampered root development in mutant mice, resulting in a shorter tooth root with a thin and hypo-mineralized root dentin, associated with premature odontoblast differentiation (51). Moreover, the deletion of Axin2, the negative regulator of the canonical pathway, also shows denser root dentin [Figure 3D; (38)].

These findings indicate that the local regulation of Wnt signaling is essential for cellular differentiation and matrix synthesis throughout the postnatal tooth root formation and root morphogenesis. Furthermore, the temporospatial stage-specific regulation of Wnt/β-catenin signaling is required in both the epithelial and mesenchymal cells for tooth root development to occur properly, as it plays an important role in the structural integrity of HERS, promotes odontoblast differentiation, and induces dentin synthesis. The canonical Wnt pathway is modulated through negative regulators, including Dkk1 and Axin2 for the proper amount of expression necessary for normal tooth root formation (Figure 3A). However, many previous studies which used a constitutively active cre system may not provide a definitive answer to tooth root formation, due to the limitation of this system to distinguish precursor-descendant relationships in a time-specific manner. Therefore, further investigation utilizing more sophisticated systems, such as tamoxifen-inducible *creER* approaches, may be needed to elucidate the detailed mechanism underlying tooth root formation.

## WNT SIGNALING AND PERIODONTIUM

Periodontium is a tooth-supporting apparatus including periodontal ligament (PDL), cementum, gingiva, and alveolar bone (52, 53). These tooth-anchoring cells originate from dental follicle progenitor cells (54). Canonical Wnt signaling is active in the periodontal complex and regulates the formation of the cementum and the periodontal ligament. Active β-catenin signals, along with Wnt10 and Axin2 expression are observed in the region surrounding the tooth root, including the HERS, dental follicle, and periodontal ligament (40, 55-57). The β-catenin expression can be found in the periodontal ligament at P14 in mice and continues to be detected in adulthood (41). Moreover, the expression of Wnt3a is found in the cellular cementum, but not in the acellular cementum. Wnt3a has an important role in inducing canonical Wnt signaling in dental follicle cells. Furthermore, Dkk2 is strongly expressed by dental follicle cells (58). Non-Canonical Wnt signaling has been found to be present in the periodontal tissue during development. Postnatally, Wnt5a is expressed in the alveolar bone, ameloblasts, odontoblasts as well as in the periodontal tissue (59, 60). Moreover, Wnt5a is co-localized with *Ocn* in root-lining follicle cells, which suggests that Wnt5a is present in precementoblasts and cementoblasts (61). Ror2 expression is detected in the periodontal and alveolar bone region in P12.5 in mice (44). It has been reported that Wnt5a expression in human PDL cells is upregulated in response to mechanical stress. Wnt5a plays important role in PDL proliferation and collagen production, by increasing periostin expression through TGFβ1 (59).

Wnt signaling regulates the proliferation and differentiation of cementoblasts, along with the development of the periodontal ligament (Figure 3A). The reduction of canonical Wnt signaling negatively affects cementoblast differentiation and cementogenesis, resulting in thinner cementum (Figure 3C). The inactivation of Wnt/β-catenin signaling, through the cell-specific ablation of β-catenin or Wntless genes in osteocalcin positive cells (*Ocn-cre; Ctnnb1^fl/fl^, Ocn-cre; Wls^fl/fl^*), disrupts the differentiation of cementoblast and reduces cementum secretion. This specific inactivation of canonical Wnt signaling also compromises collagen synthesis and fibrogenesis, resulting in reduced *Col1a1* expression in the periodontium. The absence of Wnt secretion in osteocalcin-producing cells significantly widens the periodontal ligament space with the disorganized periodontal ligament and defective collagenous extracellular matrix (49, 55). In addition, the cells in the periodontal space express fewer osteogenic markers, such as *Runx2*, *Osterix*, and *Alkaline phosphatase (ALP)*, suggesting that Wnt signaling has a role in PDL mineralization (55). The loss of β-catenin function in Gli1^+^ cells (PDL and cementum progenitor cells) (*Gli1-cre^ER^; R26R^DTA/+^; R26R^tdTomato/+^, Gli1-cre^ER^; Ctnnb^fl/fl^; R26R^tdTomato/+^*) induces a sharply decreased thickness of the cellular and acellular cementum after 3 weeks of induction (62). Moreover, the downregulation of Wnt signaling *via* Dkk1 overexpression leads to a reduced number of acellular cementoblasts with increased periodontal space, resulting from the overexpression of osteoclasts (38). Furthermore, the deletion of canonical Wnt signaling in mesenchymal cells may progressively cause root resorption due to defective root dentin and cementum, along with abnormally high osteoclast activities due to the loss of osteoprotegerin (OPG) expression (63, 64).

On the other hand, studies have shown that the overexpression of Wnt/β-catenin signaling in the mesenchymal cells, either by the constitutive activation of the pathway or the inhibition of the Wnt antagonist, can induce hyper-cementogenesis and narrow the periodontal space (Figure 3D). The persistent stabilization of β-catenin in the dental mesenchymal cells by constitutively activating β-catenin (*Ocn-cre; Ctnnb*^*lox*(*ex*3)/+^, *Col1a1-cre; Ctnnb*^*lox*(*ex*3)/+^) or by homozygously deleting *Axin2*, a negative regulator of Wnt signaling, expedites cementoblast differentiation, induces excessive cellular cementum formation, and increases *Col1a1* expression in cementoblasts and periodontal ligaments with narrow periodontal space. (41, 51, 64). The constitutive stabilization of canonical Wnt signaling in *Gli1*^+^ progenitor cells (*Gli1-creER; Ctnnb*^*flox*(*Ex*3)/+^; *R26R*^*tdTomato*/+^) in the periodontal complex shows a similar cementum hyperplasia phenotype (62). The stabilization of the β-catenin expression in osteocytes and cementocytes with *Dmp1-cre* (*Dmp1(8kb)-cre; Ctnnb*^*lox*(*ex*3)/*lox*(*ex*3)^) amplifies Wnt signaling in the periodontium, causes an excessive cellular cementum, and an abnormally calcified periodontal ligament, eventually leading to dental ankylosis (65).

Furthermore, it has been suggested that canonical Wnt signaling can enhance osteoclastogenic differentiation from fibroblasts in human periodontal ligament; in this study, Wnt signaling activation is achieved *in vitro* by treating human PDL cells with lithium chloride (LiCl). The treated cells express more ALP and other osteogenic markers, including *Osx*, *Runx2*, and *Msx2* in a dose-dependent manner (66). However, there are some conflicting reports, which demonstrate that Wnt signaling promotes proliferation and inhibits cementoblast differentiation. The administration of LiCl or GSK-3β inhibitor *in vitro* to activate the canonical Wnt pathway inhibits alkaline phosphatase activities, along with the inhibition of *bone sialoprotein (BSP)* and *osteocalcin (OCN)*, indicating the inactivation of cementoblast differentiation and reduction of mineralization. Moreover, Wnt3a stimulates cell proliferation when cells are incubated with a Wnt3a-containing conditioned medium (57).

In summary, the temporospatial regulation of Wnt signaling plays a crucial role in proper cell differentiation and periodontium formation. The Wnt pathway activity displays a biphasic function in the modulation of cementoblast differentiation, cementogenesis, and periodontal ligament formation, based on the stage of tooth development. Most studies reported that Wnt signaling positively modulates cementoblast differentiation, cementum secretion, and periodontal ligament synthesis, while the other negative regulators, such as Axin2, play critical roles in controlling the appropriate amount of Wnt expression for cementum and periodontium during tooth root development. Since most of the reports also used constitutively active *cre* system, further studies are needed to thoroughly elucidate the stage-specific mechanism of Wnt signaling in mesenchymal progenitor cells differentiation and its effect on cementogenesis.

## WNT SIGNALING IN TOOTH ERUPTION AND ALVEOLAR BONE FORMATION

The factors required for a normal tooth eruption pattern can be divided into two parts: (1) the proper amount of bone resorption and bone formation, and (2) appropriate differentiation and development of cells derived from the dental follicle mesenchymal progenitor cells (8). Wnt/β-catenin signaling has significant roles in osteoblast differentiation, bone formation, and bone homeostasis (63, 67). Wnt signaling occurs to be downstream of Bone morphogenetic proteins (BMPs) and Hedgehog (Hh) signaling, pathways known for the modulation of osteoblast linage differentiation and bone formation (67, 68). T-cell factor (TCF) is expressed in osteoblasts both during prenatal to postnatal skeletal development, suggesting active Wnt signaling activities in differentiated osteoblasts (63). Canonical Wnt signaling is required in mesenchymal precursor cells to differentiate into odontoblasts and promote the differentiation of progenitor cells into osteoblasts instead of chondrocyte and adipocytes (69, 70). In addition, Wnt signaling regulates osteoclast formation by controlling the OPG expression in osteoblast-lineage cells. The canonical Wnt signaling induces OPG expression in differentiated osteoblasts, which hinders the action of the key osteoclastogenic factor receptor activator of NF-κB ligand (RANKL) [Figure 3A; (63, 70)]. Studies have reported a direct biphasic effect of Wnt signaling in osteoclastogenesis. The activation of β-catenin is found to induce osteoclast progenitor cell proliferation in the early stage, followed by the inactivation of β-catenin to induce osteoclast differentiation (71). In addition, Wnt3a signaling also suppressed osteoclast differentiation *via* canonical and non-Canonical cyclic adenosine monophosphate (cAMP)/protein kinase A (PKA) mechanisms (72). As a result, the gain of the function mutation of β-catenin leads to osteopetrosis, or excess bone formation, from osteoclast dysfunction, while loss of function mutation results in osteoporosis from osteoclast hyperactivity (63).

Alterations of Wnt signaling directly or indirectly affect tooth eruption. The deletion of canonical Wnt signaling in odontoblasts and osteoblasts results in a normal tooth emergence pattern, despite the lack of tooth roots [Figure 3C; (48)]. The loss of *Wntless* genes in osteoblasts (*Ocn-cre; Wls^fl/fl^*) severely reduces bone mineral density and impacts the bone ultrastructure, by upregulating the RANKL expression and downregulating the OPG expression, causing elevated osteoclast differentiation and activity. Additionally, the deletion of β-catenin in osteoblasts also increases the osteoclast function and number without any effect on the bone formation, resulting in lower bone mass (38, 64). Thus, the loss of canonical Wnt signaling in osteoblast and odontoblast exhibits normal tooth eruption, even with impaired tooth root formation due to the excessive osteoclast activities with normal bone formation (Figure 3C).

Multiple studies demonstrate that excessive Wnt/β-catenin signaling impedes tooth eruption (Figure 3D). Constitutively active Wnt/β-catenin signaling pathway in odontoblasts and osteoblasts results in increased alveolar bone mass and delayed tooth eruption. The specific activation of β-catenin in odontoblasts and osteoblasts, either with *Osteocalcin* or *Col1a1* promoters, delays tooth eruption (41, 47, 51, 63, 65). These phenotypes occur as a result of the inhibition of osteoclasts. Moreover, various gene-targeted animal models with osteoclast dysfunction exhibit osteopetrosis along with failure in tooth eruption. Thus, excessive alveolar bone and defective tooth eruption phenotypes may occur due to the dysfunction of osteoclasts (41, 51, 63, 73).

Wnt5a activates the non-Canonical pathway through the Ror protein and is highly involved in bone formation and osteoclastogenesis. Wnt5a expression can be observed in osteoblast lineage cells, whereas Ror2 is observed in osteoclast precursors (74). In postnatal mice, Wnt5a protein is expressed robustly in the alveolar bone. Wnt5a expression is elevated during tooth eruption, specifically from P3 to P11 (60). Wnt5a-Ror2 signals enhance c-Jun N-terminal kinase (JNK) and recruits c-Jun promotor to gene encoding RANK, thereby inducing osteoclastogenesis (74). Moreover, according to *in vitro* study, the overexpression of Wnt5a in rat dental follicle cells reduces cell proliferation and osteogenesis. Osteogenesis markers, including alkaline phosphatase, Ocn, Runx2, and Col1a1 are increased when dental follicle cells are treated with Wnt5a protein. This result suggests that Wnt5a has a role in the differentiation and mineralization of dental follicle cells (60). Further studies are needed to shed light on the roles of non-Canonical Wnt signaling on tooth eruption and alveolar bone development.

From these previous studies, it is conceivable that the Wnt signaling in osteoblasts controls tooth eruption indirectly by modulating osteoclast differentiation and function. In the presence of Wnt/β-catenin signaling, the OPG expression in differentiated osteoblast is elevated, which inactivates RANKL expression and inactivates osteoclast function. Resultingly, the overexpression of canonical Wnt signaling disturbs osteoclast function. These over-activated β-catenin mice exhibit delayed tooth eruption with excess bone formation.

## POTENTIAL CLINICAL IMPLICATION OF WNT SIGNALING

The role of Wnt signaling in tooth development can also serve as a solid foundation for clinical implication and regenerative dentistry. Accumulating evidence suggests the potential role of this pathway in dental, periodontal, and bone regeneration. The amplification of Wnt signaling, using Axin2-deficient mice (*Axin2^LacZ/LacZ^*), is found to improve the pulp reparative response to injury by reducing cell apoptosis and promoting cell proliferation and differentiation (75). Moreover, accelerated bone healing is exhibited in Axin2-deficient mice (*Axin2^LacZ/LacZ^*), as well as in liposomal Wnt protein-injected mice. The improved bone healing is likely due to the increased proliferation and differentiation of skeletal progenitor cells (76). Furthermore, Wnt proteins can be incorporated within autografts to enhance the bone-forming capacity (77). Lastly, the activation of this signaling pathway, by deleting the Sclerostin gene, a local administration of Sclerostin antibody, and LiCl treatment, is found to enhance cementum and PDL formation in the rat periodontal defect model (78, 79). These findings illustrate the promising therapeutic potential of Wnt signaling modulation in regenerative dentistry in the future.

## CONCLUSION

Wnt signaling has a crucial role in several stages of tooth development. Wnt signaling activities are observed in the dental epithelium and the dental mesenchyme starting from the embryonic stage. In prenatal tooth formation, this pathway governs tooth number, size, and position. The lack of canonical Wnt signaling impedes tooth development, while overexpression of the pathway causes ectopic tooth formation (Table 1). Postnatally, an optimal level of Wnt signaling activities is required for normal tooth root formation and periodontal tissue formation. Wnt signaling induces odontogenesis, dentin secretion, cementogenesis, and periodontal formation, while negative regulators like *Axin2* and *Dkk1*, provide negative feedback to fine-tune the signaling pathway. The inactivation of Wnt/β-catenin signaling results in the lack of or short tooth root with thin cementum and increased periodontal space, whereas constitutive stabilization of the signal leads to a short root with thick cementum and narrow periodontal space. Moreover, this conserved signaling pathway also has significant functions in controlling bone formation and resorption, which indirectly influences tooth eruption. Wnt signaling controls bone volume and architecture through the modulation of osteoblastogenesis and osteoclastogenesis. Osteoclasts play important role in creating the path of tooth eruption. Resultingly, the constitutive activation of Wnt signaling causes osteoclast dysfunction and eventually leads to the failure of tooth eruption (Table 2, Figure 3). The increased understanding of the role of Wnt signaling in tooth development has led to the discovery that Wnt signaling may present a potential therapeutic target for disorders involving tooth root formation and tooth eruption. Further research on this subject will strengthen our understanding of the signaling pathway necessary for tooth formation and will serve as a blueprint for regenerative dentistry in the future.

## Figures and Tables

**TABLE 1 ∣:** Mouse models used for Wnt signaling role in prenatal tooth development.

Mouse genotype	Cre- promotor	Target cells	Effects on Wnt signaling	Phenotype			References
				Crown development	Crown morphology	Tooth number	
*K14-Cre;Grp117^fl/fl^*	Keratin14-Cre	Epithelial cells	Loss of function	Arrest at cap stage			(37)
*Prx1-Cre;Ctnnb1^fl/fl^*	Prx1-Cre	Dental Mesenchyme	Loss of function	Arrest at bud stage		Two incisors	(35)
*Wnt1-Cre;Lef1^fl/fl^*	Wnt1-Cre	Cranial neural crest progenitors	Loss of function	Arrest at late bud stage			(36)
*K5-rtTA tetO-Dkk1*	Keratin5-Cre	Oral ectoderm, Tooth bud	Loss of function	Arrest at early bud stage	Blunt molar cusps (When inhibited from early bell stage)		(30)
*K14-Cre;Ctnnb1^fl/fl^*	Keratin14-Cre	Epithelial cells	Loss of function	Arrest at early bud stage	Large malformed tooth bud		(30)
*K14-Cre;Ctnnb1* ^(*Ex*3)*fl*/+^	Keratin14-Cre	Epithelial cells	Gain of function	Irregular crown shape		Ectopic epithelial invagination	(30)
*K14-Cre;Ctnnb1* ^(*Ex*3)*fl*/+^	Keratin14-Cre	Epithelial cells	Gain of function	Delayed crown development	Abnormal tooth buds shape, but normal in size		(28)
2.3kb Col1a1-Dkk1	2.3kb Col1a1	Osteoblast	Loss of function		Reduced crown size	Missing third molar	(38)
		Odontoblast			Enlarged pulp		
*Dermo1-Cre;Ctnnb1* ^(*Ex*3)*fl*/+^	Dermo1-Cre	Embryonic mesenchyme					(39)
*Ors2-iresCre;Ctnnb1^fl/fl^*	Ors2-iresCre	Developing palatal and tooth mesenchyme	Loss of function	Arrest at bud to cap stage			(27)
*Ors2-iresCre;Lef^fl/fl^*	Ors2-iresCre	Developing palatal and tooth mesenchyme	Loss of function	Arrest at late bud stage			(27)
*Ors2-CreK1;Ctnnb1* ^(*Ex*3)*fl*/+^	Ors0-CreK1	Developing palatal and tooth mesenchyme	Gain of function	Progress to bell stage		Ectopic epithelial invagination at palatal shelf	(27)
				Impaired ameloblast differentiation			
				Premature differentiated odontoblast Defective ameloblast differentiation	Dentin-liked matrix in pulp		
				Defective odontoblast differentiation	Thinner dentin		
*Wnt5a* ^−/−^	Wnt1-Cre	Global knockout	Loss of function	Delayed crown development	Smaller crown size		(29)
				Reduced cell proliferation	Blunt molar cusp and incisor		
				Delayed odontoblast differentiation	Lack of Lingual 1 and Distal cusp Lack of predentin formation		
*Ror2* ^−/−^		Global knockout	Loss of function	Delayed crown development	Smaller crown size		(29)
				Defective ameloblast differentiation	Lack of predentin formation		
				Defective odontoblast differentiation	Shorter incisor		

**TABLE 2 ∣:** Mouse models used for Wnt signaling role in postnatal tooth development.

Mouse genotype	Cre- promotor	Tamoxifen injection time	Target cells	Effects on Wnt signaling	Phenotype					References
					Crown	Root	Periodontium	Bone	Tooth eruption	
*Shh-Cre^ERT2^; Ctnnb1^fl/fl^*	Shh-Cre*^ERT2^*	P4	Epithelial cells	Loss of function		Thinner HERS				(46)
						Premature dissociation of HERS Shorter molar roots				
*Shh-Cre^ERT2^; Ctnnb1* ^(*Ex*3)*fl*/+^	Shh-Cre*^ERT2^*	P4	Epithelial cells	Gain of function		Delayed disassociation of HERs				(46)
*K14-Cre;Wnt10a^fl/fl^*	K14-Cre	–	Epithelial cells	Loss of function	Taurodontism with no root furcation					(43)
*EIIa-Cre;Wnt10a^fl/fl^*	EIIa-Cre	-	Whole tissue			Shorter molar roots				
*2.3-kb Col1a1-Dkk1-Tg*	2.3kb Col1a1-cre	–	Osteoblast	Loss of function	Smaller in size	Shorter molar roots	Decreased acellular cementum	Mild bone loss		(38)
			Odontoblast		Enlarged pulp chamber	Enlarged root canal	Expanded PDL space	Increased osteoclast numbers and activities		
					Thinner dentin (from reduced dentin apposition) Immature odontoblast Reduced number and disorganized dentinal tubules					
*2.3-kb Col1a1-Cre^ERT2^; Ctnnb1* ^(*Ex*3)*fl*/+^	2.3kb Col1a1-cre*^ERT2^*	P3-P5	Odontoblast	Gain of function	Shorter incisor	Shorter molar roots	Hypoplastic cementum	Increased bone formation	Delayed tooth eruption	(47)
			Osteoblast		Premature odontoblast differentiation	Narrow PDL space	Impaired osteoclast activities			
			Cementoblast			Thin root dentin Enlarged root canal				(51)
*Ocn-Cre; Ctnnb1^fl/fl^*	1.3kb Ocn-Cre	–	Dental mesenchyme	Loss of function	Disrupted odontoblast differentiation	Calcified tissue found in PDL space	Increased osteoclast activities	Normal tooth eruption		(48)
						Rootless molar	Poorly developed periodontal structure Disrupted cementoblast differentiation Thinner cementum			(49)
*Ocn-Cre; Wls^fl/fl^*	1.3kb Ocn-Cre	–	Dental mesenchyme	Loss of function	Thinner dentin	Shorter molar roots	Thinner cementum	Increase osteoclast activities		(14)
					Enlarged pulp chamber	Thinner root dentin	Narrow PDL space	Reduced bone mineral density		(55)
					Disrupted odontoblast differentiation	Disorganized PDL				
						Root resorption				
*Gli1Cre*^*ERT2*/+^;*R26R*^*DTA*/+^;*R26R*^*tdTomato*/+^	Gli1-Cre^*ERT*2/+^	P21 and P22	Dental mesenchyme	Loss of function			Decreased cellular and acellular cementum			(62)
*Gli1Cre*^*ERT2*/+^;*Ctnnb1^fl/fl^*;*R26R*^*tdTomato*/+^		P21 and P22								
*Gli1Cre*^*ERT2*/+^;*Ctnnb1*^*lox*(*Ex*3)/+^;*R26R*^*tdTomato*/+^	Gli1-Cre^*ERT*2/+^	P21 and P22	Dental mesenchyme	Gain of function			Excessive cellular cementum			(62)
*Ocn-Cre; Ctnnb1* ^(*Ex*3)*fl*/+^	1.3kb Ocn-Cre	–	Dental mesenchyme	Gain of function	Premature odontoblast differentiation	Premature cementoblast differentiation	Impaired osteoclast activities	Delayed tooth eruption		(50)
					Thicker dentin	Shorter molar roots	Excessive cellular cementum			(41)
					Narrow pulp chamber	Thicker, but hypomineralized dentin	Narrow PDL space			
					Pulp stones found in pulp		Increased Col1a1 expression			
*DMP1-8kb-Cre;Ctnnb1* ^*lox*(*ex*3)/*lox*(*ex*3)^	DMP1-Cre	–	Osteocyte	Gain of function			Excessive cellular cementum		Delayed tooth eruption	(65)
			Cementocyte				Calcified PDL Ankylosis			
*Osr2-Cre; Ror2^fl/fl^*	Osr2-Cre	–	Dental mesenchyme	Loss of function		Shorter molar roots				(44)
					Disrupted odontoblast differentiation					
